# Novel *PHOX2B* germline mutation in childhood medulloblastoma: a case report

**DOI:** 10.1186/s13053-021-00170-5

**Published:** 2021-01-19

**Authors:** Caiping Ke, Xiaoshun Shi, Allen Menglin Chen, Chaoming Li, Bifeng Jiang, Kailing Huang, Zhouxia Zheng, Yanhui Liu, Zhuona Chen, Yingjun Luo, Huaming Lin, Jiexia Zhang

**Affiliations:** 1 First Tumor Department, Maoming People’s Hospital, Maoming, 525000 China; 2 Department of Thoracic Surgery, Nanfang Hospital, Southern Medical University, Guangzhou, 510515 People’s Republic of China; 3 Guangzhou Mendel Genomics and Medical Technology Co., Ltd., Guangzhou, 510535 China; 4 Mendel Genes Inc, Manhattan Beach, CA USA; 5 State Key Laboratory of Respiratory Disease, National Clinical Research Center for Respiratory Disease, Guangzhou Institute of Respiratory Health, the First Affiliated Hospital of Guangzhou Medical University, Guangzhou, 510120 China

**Keywords:** Medulloblastoma, *PHOX2B*, Germline mutation, Whole exome sequencing, Cancer screening

## Abstract

**Background:**

Medulloblastoma is an aggressive brain tumor mostly found in children, few studies on pathogenic germline mutations predisposing this disease was reported.

**Case presentation:**

We present an 11-year-old male with medulloblastoma, who harbors a de novo *PHOX2B* germline mutation as detected by whole exome sequencing (WES). Family history was negative. Sanger sequencing confirmed this mutation in peripheral blood, hair bulbs, urine and saliva. Identification of novel germline mutations is beneficial for childhood cancer screening.

**Conclusions:**

This case revealed a de novo *PHOX2B* germline mutation as a potential cause of medulloblastoma in a child and suggests familial germline variant screening is useful when an affected family is considering having a second child.

**Supplementary Information:**

The online version contains supplementary material available at 10.1186/s13053-021-00170-5.

## Introduction

Medulloblastoma is a malignant tumor of the cerebellum that is most common in childhood, characterized by highly malignant manifestations including rapid tumor growth, high recurrence rate, and poor overall survival [1]. Large-scale genetic studies have revealed somatic and germline mutations that associated with the disease. One genetic study showed that *KBTBD4* and *PRDM6* are candidate driver mutations in medulloblastoma [2]. In addition, six germline mutations: *APC*, *BRCA2*, *PALB2*, *PTCH1*, *SUFU*, and *TP53*, were reported to be responsible for 6% of medulloblastoma cases [3]. However, these mutations may not be able to fully explain the susceptibility and pathogenesis of a sporadic case.

*PHOX2B* encodes neuroblastoma Phox (paired-like homeobox 2B) protein, which plays a role in neuron development and involves in the determination of the neurotransmitter phenotype. It is reported to be associated with congenital central hypoventilation syndrome [4] and hereditary neuroblastic tumours [5]. The pathogenic roles of *PHOX2B* mutations have been published in the ClinVar database, but few reports exist on de novo germline mutations associated with childhood medulloblastoma development. By using whole exome sequencing (WES, the NovaSeq 6000 Sequencing System, Illumina) technology and Sanger sequencing validation, we report the case of a child with a de novo c.765_779 deletion of *PHOX2B* as a contributor to the risk of medulloblastoma.

## Methods and results

### Case descriptions

**Fig. 1:**
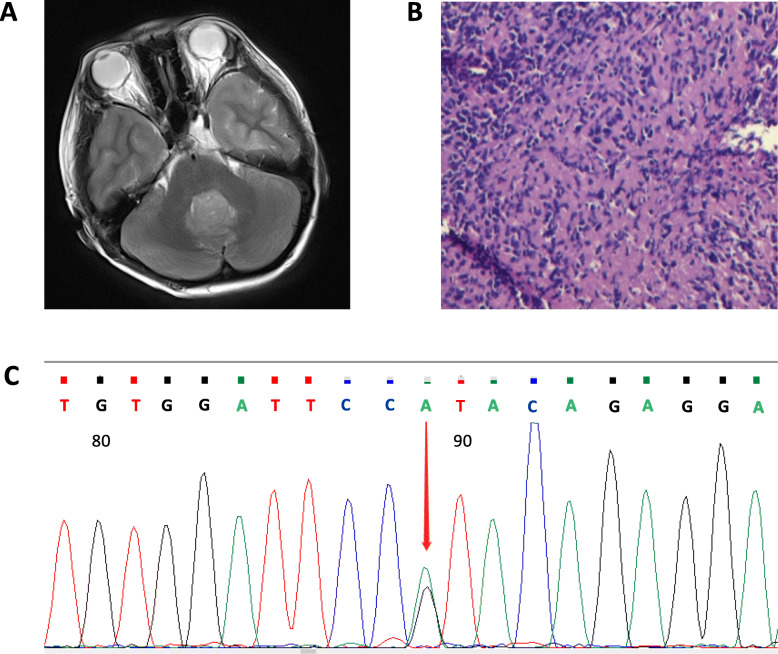
The discovery of a novel *PHOX2B* mutation. **a** MRI imaging indicating a 30 mm X 30 mm X 21 mm solid tumor at the fourth ventricle; **b** Pathological findings of surgical tissue; **c** Electropherogram showing the c.765_779del mutation in *PHOX2B*. Other electropherogram images across tissues are list in supplementary Table 1

An 11-year-old male patient who had an accidental fall in December 2018 with no other past medical history was seen in our hospital. Subsequent head and whole spinal cord MRI showed lesions in the fourth ventricle, suggesting a likelihood of medulloblastoma (Fig. 1a). On 2019-01-10, following general anesthesia, a cranial fossa craniotomy, cerebellar tumor resection, dural repair, and decompressive craniectomy were performed. After the surgical treatment and five cycles of temozolomide, the patient is stable. Postoperative pathology diagnosis was cerebellum medulloblastoma (WHO-IV). Immunohistochemistry showed Vimentin (−), CK (−), GFAP (−), S-100 (+/−), KI67 (30% +), P53 (−), CD99 (−), CD56 (+), SYN (+), and NSE (+) (Fig. 1b).

The patient’s parents were concerned a second child might be affected. Therefore, genetic testing was done in which three mutations were detected, including a c.505A > G point mutation in the *MSH2* gene (NM_000251 transcript), a c. 6139A > G point mutation in the *MED12* gene (NM_005120 transcript) and a c.765_779del deletion mutation in the *PHOX2B* gene (NM_003924 transcript). The c.505A > G point mutation and the c.765_779del deletion were heterozygous mutations, while the c.6139A > G point mutation was a homozygous mutation. According to the ClinVar database, the c.505A > G point mutation in the *MSH2* gene is a possible benign variation (Likely benign), the c.6139A > G point mutation in *MED12* is of unknown clinical significance, and the c.765_779del deletion mutation of *PHOX2B* is Benign/Likely benign. Further familial genetic testing showed that the c.505A > G point mutation of *MSH2* and the c.6139A > G point mutation of *MED12* were inherited from his mother. Of note, the c.765_779del deletion mutation of *PHOX2B* was a de novo mutation (Fig. 1c). These germline mutations were confirmed by Sanger sequencing on samples obtained from patient’s peripheral blood, saliva, hair, and urine. We also conducted DNA paternity testing to confirm that the parents are the patient’s biological parents (Table 1, supplementary Table 1).

**Table 1:** Detected germline variants in patient and his parents

Gene	Transcript	Nucleotide change	Amino acid change	Mutation frequency	ACMG grade	Patient (Blood)	Patient (Tissue)	Patient (Saliva)	Patient (Urine)	Father	Mother	OMIM/ClinVar
*MSH2*	NM_000251	chr2:47637371 c.A505G (rs63750716)	p.I169V	0.006361	likely benign	heterozygous	heterozygous	heterozygous	heterozygous	no mutation	heterozygous	Lynch syndrome, Turcot syndrome Mismatch repair cancer syndrome [6]
*MED12*	NM_005120	chrX:70360579 c.A6139G (rs748668603)	p.I2047V	0.001032	uncertain significance	homozygous	homozygous	homozygous	low contentration,unable to detect	no mutation	heterozygous	Lujan-Fryns syndrome [7], Ohdo syndrome, Opitz-Kaveggia syndrome
*PHOX2B*	NM_003924	chr4:41747990–41,'748,004 c.765_779del (rs761018157)	p.255_260del	–	benign / likely benign	heterozygous	heterozygous	heterozygous	low contentration,unable to detect	no mutation	No mutation	Neuroblastoma [8], Hirschsprung disease

### Sample pre-processing

DNA was extracted from 1 ml of peripheral blood by a Blood genomic DNA Mini Kit (CW2087, Cwbio, China), at least 5 hair bulb and 35 ml urine by the universal genomic DNA Kit (CW2298, Cwbio, China), and 0.8 ml saliva by the CW2655 kit (CW2655, Cwbio, China), according to the manufacturer’s instructions. The extracted DNA was dissolved in 100 μl TE buffer, quantified using a NanoDrop spectrophotometer and stored at − 80 °C until use. The Medical Ethics Committee of the Maoming People’s Hospital reviewed and approved this study. Both parents and the patient signed an informed consent. No personal information will be disclosed in this study.

### PCR amplification and sanger sequencing

DNA was amplified using specific primers listed below (Table 2). PCR amplification was performed using the following cycle conditions: pre-denaturing at 95 °C for 1 min; 45 cycles consisting of 95 °C for 45 s, 57 °C for 45 s, 68 °C for 1 min; and final extension at 68 °C for 3 min. The PCR products were analysed on a 1% agarose gel. Sequences reactions were run on an ABI 3130xl Genetic Analyzer (Applied Biosystems, Life Techologies, Carlsbad, CA, USA) following the manufacturer’s instructions. Sequences were analysed with Mutation Surveyor software (Softgenetics, State College, PA, USA) using human genome hg19 as reference.

**Table 2:** Primers design

Gene	Exon	Forward primer	Reverse primer
MSH2	Exon3	GATATGTCAGCTTCCATTGGTGTTG	GGCCTGGAATCTCCTCTATCACTA
MED12	Exon42	CAGGTCAGGGACCCAAGGTTTATAC	CAATGTCCAACTCTCTCCCACTAT
PHOX2B	Exon3	CAGATCAGAACATACTGCTCTTCACT	GCCAAGTTTCGCAAGCAGGAG

### Paternity testing

As the Goldeneye™ 20A system exhibited a robustness to a level of forensic biological evidence, the DNA identification was used the Goldeye™ 20A kit (Peoplespot Inc. Beijing, China) following the instructions of the manufacturer. Data analysis such as allelic typing was performed using Gene Marker HID software.

The cumulative parental index (CPI) is defined as:

$$ \mathrm{CPI}={\sum}_{\mathrm{i}=1}^{\mathrm{n}}\mathrm{PIi}, $$

Where the paternity index (PI) is: PI = X/Y, PIi is the paternity index PI when the short tandem repeat (STR) locus is i.

The relative probability of paternity (RPP) = CPI / (CPI + 1)) × 100%.

In this case, the detection of 19 autosomal STR loci revealed that the mother and child were in full compliance with Mendel’s law of inheritance at these 19 STR loci, and that the father and child were also in line with Mendel’s law of inheritance at these 19 STR loci. The CPI was 4.5*10 [9], confirming the biological parental relationship.

## Discussion

The *PHOX2B* gene encodes paired-like homeobox 2b protein, which is expressed in the nervous system. Clinically, immunohistochemical staining of *PHOX2B* protein is a sensitive and specific marker for undifferentiated neuroblastoma [9, 10]. In addition, mutations of *PHOX2B* gene, both somatic [11] and germline [5], have been reported in previous neuroblastoma studies. In most cases, these mutations are somatic mutations while germline mutations inherited from the patient’s parents are less common. Founder germline mutation of *PHOX2B* that cause childhood medulloblastoma are even more rare. Here, we identified the c.765_779del deletion of *PHOX2B* in a patient with medulloblastoma and confirmed that the mutation existed in other tissues from the patient. However, the mutation was absent from his biological parents. Previous studies showed that *PHOX2B* is associated with neuroblastoma. Meanwhile, our data suggest that the c.765_779del deletion serves as a potential de novo germline mutation that causes medulloblastoma. Further biomolecular studies on *PHOX2B* are necessary for better understanding its pathogenic role in medulloblastoma.

Recent evidence showed that de novo mutations contribute to a genetic source of cancer causality. Chompret et al. reported that de novo mutations of p53 in childhood cancer are not rare [12]. In addition, a de-novo splice site mutation c.2006-2A > G in the *MSH2* gene was found in a young colon cancer patient with negative family history [13]. Paola et al. reported that 38G > A (G13D) is a de novo mutation of *NRAS* responsible for juvenile myelomonocytic leukaemia [14]. Based on these findings and the purpose of genetic testing in this case (considering having a second child), pathogenic variant screening of parents is informative for making second child decisions. Moreover, in order to aim at better clinical management, it is necessary to well document similar cases and to analyse the difference between novel mutated cases and ordinary cases in terms of pathogenesis, disease development, degrees of clinical severity and prognosis.

## Conclusion

In this case, we reported a de novo *PHOX2B* germline mutation as a potential cause of medulloblastoma in a child. Familial germline variant screening is a recommended tool when an affected family is considering having a second child.

## Supplementary Information

**Additional file 1.**

## Data Availability

All data generated or analysed during this study are included in this published article and its supplementary information files.

## Abbreviations

WES: Whole exome sequencing
PCR: Polymerase chain reaction
CPI: Cumulative parental index
PI: Paternity index
STR: Short tandem repeat
RPP: Relative probability of paternity
